# Pilot proof of concept clinical trials of Stochastic Targeted (STAR) glycemic control

**DOI:** 10.1186/2110-5820-1-38

**Published:** 2011-09-19

**Authors:** Alicia Evans, Geoffrey M Shaw, Aaron Le Compte, Chia-Siong Tan, Logan Ward, James Steel, Christopher G Pretty, Leesa Pfeifer, Sophie Penning, Fatanah Suhaimi, Matthew Signal, Thomas Desaive, J Geoffrey Chase

**Affiliations:** 1 Department of Mechanical Engineering, Centre for Bio-Engineering, University of Canterbury, Christchurch, New Zealand; 2 Department of Intensive Care, Christchurch Hospital, Christchurch School of Medicine, University of Otago, Christchurch, New Zealand; 3 Cardiovascular Research Centre, University of Liege, Liege, Belgium

## Abstract

**Introduction:**

Tight glycemic control (TGC) has shown benefits but has been difficult to achieve consistently. STAR (Stochastic TARgeted) is a flexible, model-based TGC approach directly accounting for intra- and inter- patient variability with a stochastically derived maximum 5% risk of blood glucose (BG) < 4.0 mmol/L. This research assesses the safety, efficacy, and clinical burden of a STAR TGC controller modulating both insulin and nutrition inputs in pilot trials.

**Methods:**

Seven patients covering 660 hours. Insulin and nutrition interventions are given 1-3 hourly as chosen by the nurse to allow them to manage workload. Interventions are calculated by using clinically validated computer models of human metabolism and its variability in critical illness to maximize the overlap of the model-predicted (5-95^th ^percentile) range of BG outcomes with the 4.0-6.5 mmol/L band while ensuring a maximum 5% risk of BG < 4.0 mmol/L. Carbohydrate intake (all sources) was selected to maximize intake up to 100% of SCCM/ACCP goal (25 kg/kcal/h). Maximum insulin doses and dose changes were limited for safety. Measurements were made with glucometers. Results are compared to those for the SPRINT study, which reduced mortality 25-40% for length of stay ≥3 days. Written informed consent was obtained for all patients, and approval was granted by the NZ Upper South A Regional Ethics Committee.

**Results:**

A total of 402 measurements were taken over 660 hours (~14/day), because nurses showed a preference for 2-hourly measurements. Median [interquartile range, (IQR)] cohort BG was 5.9 mmol/L [5.2-6.8]. Overall, 63.2%, 75.9%, and 89.8% of measurements were in the 4.0-6.5, 4.0-7.0, and 4.0-8.0 mmol/L bands. There were no hypoglycemic events (BG < 2.2 mmol/L), and the minimum BG was 3.5 mmol/L with 4.5% < 4.4 mmol/L. Per patient, the median [IQR] hours of TGC was 92 h [29-113] using 53 [19-62] measurements (median, ~13/day). Median [IQR] results: BG, 5.9 mmol/L [5.8-6.3]; carbohydrate nutrition, 6.8 g/h [5.5-8.7] (~70% goal feed median); insulin, 2.5 U/h [0.1-5.1]. All patients achieved BG < 6.1 mmol/L. These results match or exceed SPRINT and clinical workload is reduced more than 20%.

**Conclusions:**

STAR TGC modulating insulin and nutrition inputs provided very tight control with minimal variability by managing intra- and inter- patient variability. Performance and safety exceed that of SPRINT, which reduced mortality and cost in the Christchurch ICU. The use of glucometers did not appear to impact the quality of TGC. Finally, clinical workload was self-managed and reduced 20% compared with SPRINT.

## Introduction

Stress-induced hyperglycemia often is experienced in critically ill patients with increased morbidity and mortality [1,2] in this highly insulin resistant in this group of patients [1-7]. Glycemic variability and thus poor control [8] are independently associated with increased mortality [9,10]. Tight glycemic control (TGC) can significantly reduce the rate of negative outcomes associated with poor control by modulating insulin and/or nutrition administration [7,11,12], including reducing the rate and severity of organ failure [13] and cost [14,15]. However, safe, consistent, and effective TGC remains elusive with several inconclusive studies [16-19]. There is little agreement on the definition of desirable glycemic performance [20-22], particularly with regard to how TGC may affect outcome.

The SPRINT protocol was successful at reducing organ failure and mortality [11,13], with a patient-specific approach that directly considered carbohydrate administration along with insulin. It provided the tightest control across all patients of several large studies [8,23], via a patient-specific approach accounting for inter- and intra- patient variability in metabolic behavior. However, the protocol is relatively inflexible, and the clinical burden, although acceptable, was higher than desired. In particular, SPRINT had a fixed glycemic target of 4.0-6.1 mmol/L, fixed measurement intervals and rules, and a fixed approach with respect to the balance of insulin and nutrition. Hence, although unique in its control of nutrition as well as insulin, it had no ability to customize the glycemic target, control approach, or workload to specific patients, conditions, or responses, all of which are issues common to most TGC protocols that can hinder uptake and compliance [24-26]. Model-based approaches have been mooted as a solution [27,28].

This paper presents the initial proof of concept pilot clinical trial results for a model-based TGC protocol that ameliorates or eliminates all these issues with clinically specified glycemic targets and nurse selected measurement intervals (with associated interventions). The metabolic system model uses additional stochastic models [29,30] to forecast the range of glycemic outcomes for a given intervention, providing greater certainty over longer measurement intervals, and the ability to identify a clinically specified level of risk of exceeding clinically specified levels of hypo- or hyperglycemia. Its adaptive, patient-specific control approach is fully customizable to local clinical standards.

## Methods

### Patients

Seven patients were recruited based on the need for TGC (BG > 8.0 mmol/L) or existing treatment with SPRINT [11], the current standard of care at Christchurch Hospital. Table 1 shows the patient cohort details. Written, informed consent was obtained for all patients, and approval was granted by the NZ Upper South A Regional Ethics Committee.

**Table 1:** Baseline clinical data for STAR pilot trials patients

Patient	Age	Sex	Hours	Diagnosis	APACHE II	APACHE III
**A^a^**	61	M	92	AAA Rupture	23	117

**B^a^**	61	M	17	AAA Rupture	23	117

**C**	80	M	264	Head Trauma	16	75

**D**	80	M	96	CABG	21	85

**E**	65	F	119	Pancreatic Surgery	13	58

**F**	66	M	23	GI Surgery (post)	22	83

**G**	52	F	49	Pancreatitis	14	70

AAA = Abdominal Aortic Aneurysm; APACHE = Acute Physiology and Chronic Health Evaluation; CABG = Coronary Artery Bypass Graft; GI = Gastro-Intestinal.

^a ^Consecutive episodes of insulin usage in same person.

### Stochastic TARgeted glycemic control

**Figure 1:**
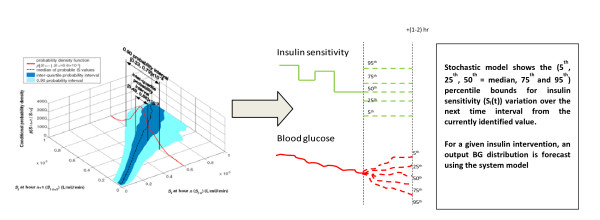
**Stochastic model (left) can be used with an identified current level of S_I_(t) to provide a forecast range of S_I_(t) values over the next 1- to 3-hour interval**. This forecast range of values can be used with a given insulin intervention and the system model of Equations (1)-(6) to yield a range of BG outcomes of differing likelihood. Note that the stochastic model shown is for a 1-hour interval, the 2- to 3-hour interval models are very similar but not shown here. More details are provided in previous studies [29,30].

The Stochastic TARgeted (STAR) TGC protocol recommends insulin and nutrition interventions based on the current patient-specific insulin sensitivity (S_I_(t)). Insulin sensitivity is identified hourly for each patient using recent BG measurements and a computerized metabolic system model. With this value, the predicted blood glucose response to a particular intervention can be calculated. A stochastic model [29,30] of the potential variability in S_I_(t) over the subsequent 1-3 hours is used to capture the potential variation of (patient-specific) modeled insulin sensitivity and thus the potential range of glycemic outcomes to an intervention. Although the median and most likely variation is no significant change from the previous hour, the interquartile range (IQR) and (5^th^, 95^th^) percentile variations can result in significant changes in BG for a given insulin intervention. The stochastic models and their use in TGC are presented in detail in references [29-32]. Figure 1 schematically shows this model and its potential use to determine the impact of variable insulin sensitivity on BG outcome for a given intervention.

The STAR approach explicitly targets the (5-95^th^) percentile outcomes shown in Figure 1 to best overlay a clinically chosen target range of 4.4-6.5 mmol/L, yielding a maximum likelihood of being in this band. The fifth percentile is never allowed to be lower than 4.0-4.4 mmol/L, providing a risk of 5% for BG below these values for any intervention. This level can be clinically specified and can be different for different measurement intervals. For every intervention, the nurses have a free choice of measurement interval of 1, 2, or 3 hours when BG is within 4.0-7.5 mmol/L with a forecasted risk of hypoglycemia within tolerance, and measured BG was not significantly below previously forecasted values. Outside this range, targeting and measurement interval are restricted to 1 hour for patient safety. Table 2 shows the target to range approach clinically specified for this study.

**Table 2:** STAR BG target ranges and approach for BG in the 4.0-7.5 mmol/L range

Measurement interval	BG percentile and target BG for that measurement interval	Goal and outcome
1-hour	95^th ^percentile is targeted equal to 6.5 mmol/L unless 5^th ^percentile BG < 4.0 mmol/L ELSE: 5^th ^percentile targeted at 4.0 mmol/L	Ensures 95% of outcome BG are in 4.0-6.5 mmol/L target range and risk of moderate hypoglycemia BG < 4.0 mmol/L does not exceed 5%.
2-hour	5^th ^percentile targeted at 4.4 mmol/L	Ensures most likely BG values are in 4.4-6.5 mmol/L range, and a maximum risk of 5% for BG < 4.4 mmol/L. It also accepts a potentially greater likelihood of exceeding 6.5 mmol/L at end of interval as preferable to being lower than 4.4 mmol/L.
3-hour	5^th ^percentile targeted at 4.4 mmol/L	Ensures most likely BG values are in 4.4-6.5 mmol/L range, and a maximum risk of 5% for BG < 4.4 mmol/L. It also accepts a potentially greater likelihood of exceeding 6.5 mmol/L at end of interval as preferable to being lower than 4.4 mmol/L.

Specific insulin and nutrition interventions are optimized using an extensively, clinically validated [33-39] system model detailed in the Appendix (Additional File 1). The model is used to identify current insulin sensitivity (S_I_(t)) and to predict outcomes (Figure 1) for different possible interventions. The discrete insulin and nutrition doses used and limits on allowed dosing changes from a prior intervention are defined in Table 3, where these limits provide robustness to assay error and patient safety.

**Table 3:** Insulin and nutrition dose increments and limits on rate of change in dose per measurement interval designed for patient safety

Intervention	Increments used	Maximum change
Insulin	0.0-6.0 U/h in increments of 0.5 U excluding 0.5 U/h	+3U (dosing is per hour) reduce to 0 U/h
Nutrition	30-100% of ACCP/SCCM goal feed of 25 kcal/kg/h [40,41] in increments of 5%, using a low carbohydrate enteral nutrition formula (local clinical standard) of 35-40% carbohydrate content. Nutrition may be turned off for other clinical reasons (0%) leaving only insulin as an intervention Same rules apply if parenteral nutrition is used	± 20% May be set to 0% if clinically specified

At each measurement, the algorithm searches over all feasible solutions within these intervention constraints. If no feasible solution is available for a 2- to 3-hour interval, the 5^th ^percentile is set for a value > 4.4 mmol/L within these limits. If more than one solution is feasible for a given measurement interval, then the algorithm selects that which is the same as, or closest to, the prior intervention to minimize clinical effort (e.g., keeping the enteral feed rate and/or insulin input the same). If both interventions are changing, then the protocol selects the feasible option with greatest nutrition administration, a choice that was clinically specified.

Finally, Table 4 defines four special cases for which measurement intervals are restricted to 1 and/or 2 hourly and interventions modified, and/or where the interventions are modified for highly insulin resistant patients where the limits of Table 3 are not sufficient to reduce hyperglycemia. Each case represents a significant risk to patient safety where insulin can be dosed excessively in other protocols. Computer-based, STAR automatically detects these situations and offers only the relevant options.

**Table 4:** Special cases definitions and outcome impact on interventions and measurement interval

Case	Condition		Outcome		Maximum measurement interval (h)
Gradual reduction of hyperglycemia	BG_i _> 7.5 mmol/L		Percentile used for Targeting	50^th^	1
			Target Value	0.85 × BG_i_	
Rapid decrease in glucose levels	BG_i _< BG_i-1 (5th) _- 1	BG_i _< 5.0	Background insulin infusions stopped		1
		BG_i _≥ 5.0	Background insulin infusions stopped		
Nutrition suspension	Feed turned off by clinician		Use only insulin intervention Stop all extra insulin infusions		2
Added insulin infusion of 1 U/h over 6 U/h maximum	Must meet: • Insulin at ≥5 U/h, for the past 3 hours • At least 4 hours has elapsed since the last time the enteral feed was turned off		Add 1 U/h insulin infusion on top of 6 U/h maximum level This infusion is maintained for 6 hours unless: A) Nutrition is stopped for any reason B) If "Rapid Decrease in Glucose Levels" is detected C) BG predicted to be below lower cceptable limit with insulin infusion		1-3 hours as chosen by nurse

Finally, it is important to note that STAR is a framework, rather than a specific protocol. The STAR framework is the overall stochastic approach to glycemic control shown in Figure 1. It includes the ability to specify risk of hypoglycemia below a clinically set threshold (Table 2), and the ability to enable multiple hourly measurements based on clinically set glycemic thresholds (Table 2). Within that framework, clinical or site-specific constraints may be added for how control is provided (Table 3), which is via insulin and nutrition control in this study with insulin delivered primarily via bolus delivery, and any special cases or rules (Table 4). Hence, STAR is a flexible framework or overall model-based approach that could admit a multitude of control approaches that could be quite different than the specifics used here. Specifically, two uses of STAR might provide very different glycemic outcomes.

### Analyses

Data are presented as median [IQR] for both cohorts and for median values across patients. For contextual comparison only, the same glycemic outcomes are shown for all 371 patients reported for SPRINT [11]. Cumulative time in the 4.0-7.0 mmol/L band over 50% (cTIB ≥ 0.5) was associated with faster reduction in organ failure in SPRINT [13] and also is assessed. Data for time in band assessments was resampled between measurements to ensure the same measurements per day for each cohort compared, so there was no bias from different measurement intervals. Safety from hypoglycemia is assessed for moderate (percent BG < 4.0 mmol/L and < 4.4 mmol/L) and severe (number with BG < 2.2 mmol/L). Finally, measurements per day and the number of unchanged interventions are recorded as surrogates for clinical effort.

## Results

Table 5 shows the glycemic control results for the cohort. Table 6 shows the glycemic control results per patient. Overall performance is similar or slightly better for STAR versus the (contextual comparison only) SPRINT data. Moderate hypoglycemia (BG < 4.0 mmol/L) is under the clinically specified threshold risk of 5%, as designed. Equally, the number of measurements per patient was reduced ~20% for the patients studied compared to SPRINT and the number of unchanged interventions was similar for the cohort. However, the per-patient results showed significant increases in unchanged interventions (Table 6), indicating that STAR was more dynamic for variable patients, as required (patient C in particular), and less so for others.

**Table 5:** Summary of cohort glycemic performance results

	STAR pilot trials	SPRINT clinical data
BG median [IQR] (mmol/L)	5.9 [5.2-6.8]	5.7 [5-6.6]
		
%BG in 4.0-6.5 mmol/L	63	70
%BG in 4.0-7.0 mmol/L	76	79
%BG in 4.0-8.0 mmol/L	90	88
		
%BG < 4.4 mmol/L	8.0	9.1
%BG < 4.0 mmol/L	4.2	3.8
		
Median insulin rate [IQR] (U/hr)	2.5 [0.0 - 6.0]	3.0 [2.0 - 3.0]
Median glucose rate [IQR] (g/hr)	6.8 [5.5-8.7]	3.8 [1.6-5.5]
		
Average measurements/day	15	15
% Unchanged enteral nutrition interventions	86%	80%
% Unchanged insulin interventions	39%	48%
% Unchanged insulin AND nutrition interventions	36%	41%

**Table 6:** Summary of per-patient glycemic performance results

	STAR pilot trials	SPRINT clinical data
Hours of control (h)	92 [29.5-113.3]	53 [19-146]
		
Median BG median [IQR] (mmol/L)	5.9 [5.8-6.3]	5.8 [5.3-6.4]
		
%BG in 4.0-6.5 mmol/L	61.1 [55.3-78.4]	66.7 [51.7-78.9]
%BG in 4.0-7.0 mmol/L	79.2 [68.6-88.8]	77.2 [63.6-86.8]
%BG in 4.0-8.0 mmol/L	96.2 [89.3-100]	86.6 [75-94.3]
		
%BG < 4.4 mmol/L	4.3 [0.4-11]	6.9 [1-16.1]
%BG < 4.0 mmol/L	0 [0-6]	1.8 [0-6.9]
		
Median insulin rate [IQR] (U/h)	2.5 [0.1-5.1]	3.0 [2.0 - 3.0]
Median glucose rate [IQR] (g/h)	6 [5.6-6.9]	2.2 [0-4.5]
		
Average measures/day	14	17
%Unchanged nutrition interventions	86 [83-93]	82 [72-90]
%Unchanged insulin interventions	59 [27-75]	42 [30-54]
%Unchanged insulin AND nutrition interventions	58 [20-74]	36 [25-48]

**Figure 2:**
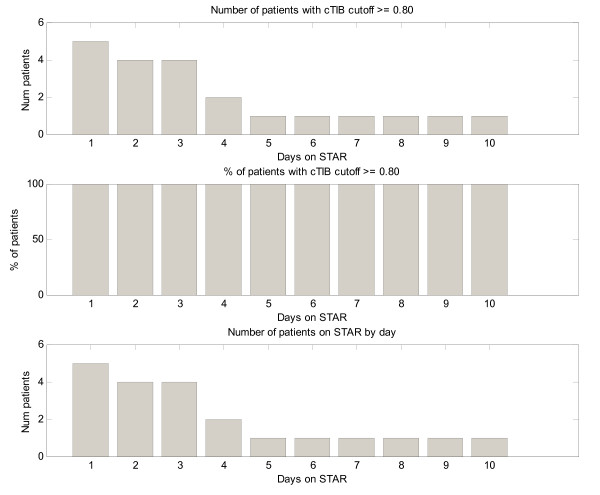
**Number and percentage of patients with cumulative time in the 4.0-7.0 mmol/L band of at least 80% per day, along with number of patients on STAR per day**.

**Figure 3:**
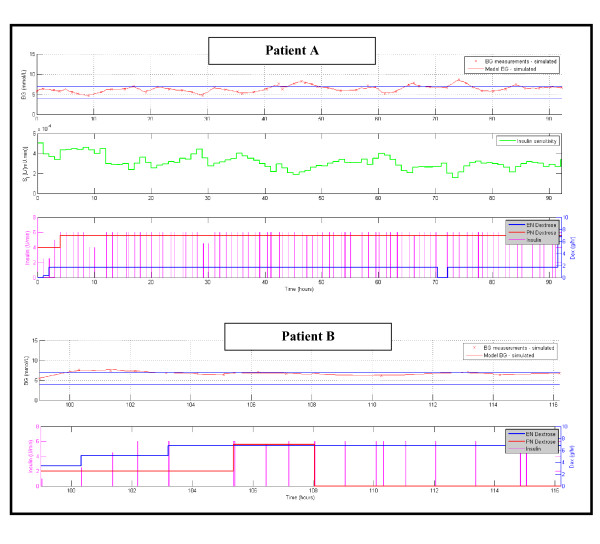
**Patients A and B, glycemic outcomes with STAR (top panel) and interventions (bottom panel)**. Patient A shows (middle panel) the model identified insulin sensitivity (SI(t), see Appendix (Additional File 1) for details). For BG, the "x" symbols are measured BG values and the solid line is the modeled value. The straight horizontal lines in the BG plots are at 4.0 and 7.0 mmol/L defining that range between them.

**Figure 4:**
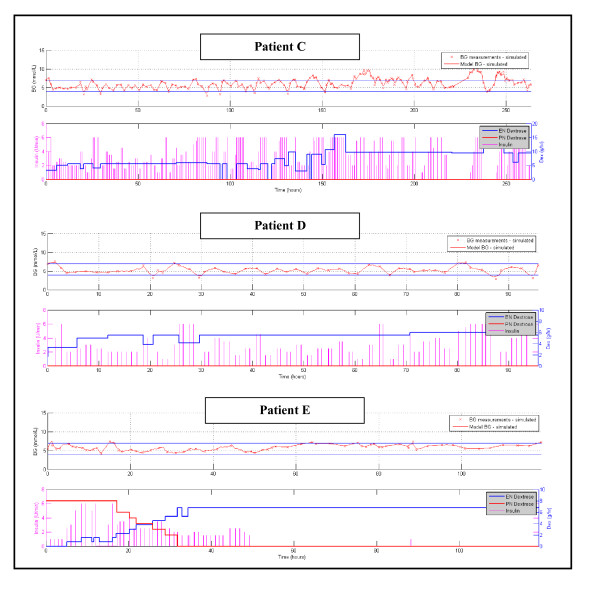
**Patients C, D, and E, glycemic outcomes with STAR (top panel) and interventions (bottom panel)**. For BG, the "x" symbols are measured BG values and the solid line is the modeled value. The straight horizontal lines in the BG plots are at 4.0 and 7.0 mmol/L defining that range between them.

**Figure 5:**
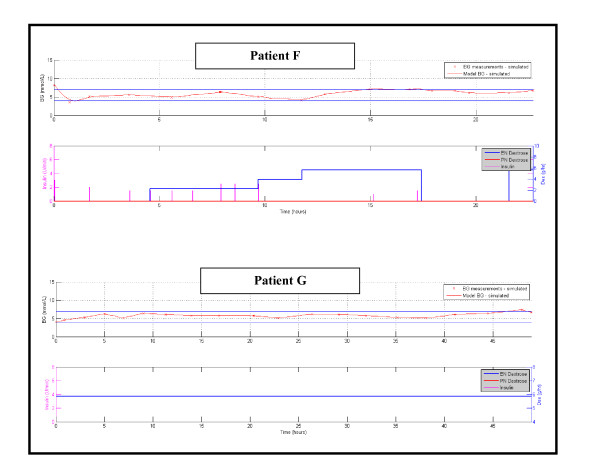
**Patients F and G, glycemic outcomes with STAR (top panel) and interventions (bottom panel)**. For BG, the "x" symbols are measured BG values and the solid line is the modeled value. Note that patient G received constant enteral nutrition rate on clinical orders and STAR managed, which change directly by recognizing that there was no need for insulin, because the patient (previously on SPRINT) was stable.

Figure 2 shows the number of patients for each day with cTIB ≥ 0.8 (band = 4.0-7.0 mmol/L, BG data resampled hourly), where all patients achieved this level for all days. Figures 3, 4 and 5 show the BG data, model curve, and interventions for all seven patients; Figure 3 also shows the modeled insulin sensitivity for patient A, which is used as input for the stochastic model (see Figure 1) to forecast the range of possible intervention outcomes in optimising interventions. Hence, control was very tight.

Also of note, patient G received a constant enteral nutrition rate on clinical orders. STAR managed that change directly and, equally importantly, recognized there was no need for insulin as the patient (previously on SPRINT) was stable. Equally, patient E became stable and did not require insulin in the second half of the trial, before STAR was stopped as a result, which also was recognized by STAR and the model as it eventuated. Thus, overcontrol and excessive insulin use was avoided.

## Discussion

STAR is a unique, model-based TGC protocol that uses clinically validated metabolic and stochastic models to optimize treatment in the context of possible future patient variation. Probabilistic forecasting enables more adaptive, optimized patient-specific care with clinically specified maximum risk(s) of hyper- and hypoglycemia. This forecasting capability is only possible in computerized, model-based protocols, and enables increased protocol flexibility, increased safety, and reduced clinical effort, in this case by design.

The stochastic approach enables a unique targeting method, where interventions are selected to maximize the likelihood of BG in a clinically specified range, while providing a clinically specified maximum acceptable risk of light hypoglycemia. The stochastic output range is thus overlaid with a clinically specified desired control range (4.0-4.4 → 6.5 mmol/L depending on intervention interval in this case) to maximize the likelihood of being in that range. Its control thus selects treatments that are justified by their predicted effect on the full range of possible BG outcomes.

To date, the initial clinical results are positive. Patients C and D, for example, clearly demonstrate different levels of intra-patient and inter-patient metabolic variability, all of which was equally well managed with respect to glycemic performance and safety. Patient E was a unique case, where the controller recognised the relatively high insulin sensitivity of the patient after about half their stay and was able to recommend no insulin be given. This recommendation was correct given the resulting good glycemic control within the desired target band for more than ~50 subsequent hours. The correct recommendation of no insulin is one that many protocols find difficult as their design is implicitly based on and biased toward active intervention. Hence, the STAR controller was able to avoid overcontrolling the patient with insulin where necessary.

The remaining four patients had similarly good results (Tables 5, 6 and Figures 3, 4, 5), particularly for achieving high cTIB ≥ 0.8 values (Figure 2), where patients had cTIB ≥ 0.8 for all days. These cTIB results indicate that control over all patients in this initial study was very tight compared with SPRINT (as seen in [13]). Thus, initially, STAR appears able to provide tighter control across patients than SPRINT, which also is seen in Table 5 and particularly in Table 6 where median values across patients are much more tightly clustered over a 0.5 mmol/L wide interquartile range.

The STAR framework and approach presented allows nurses free choice of measurement interval to reduce real and perceived clinical burden through longer intervals (compared to SPRINT) and free choice [24,26]. While longer intervals used different targeting, the overall glycemic performance was very comparable to SPRINT. Equally, all degradation or difference in control in Tables 5 and 6 was toward a moderately hyperglycemic range. This result is partly due to the higher (4.4 vs. 4.0 mmol/L) 5% maximum hypoglycemic risk threshold specified at these intervals (Table 2). This approach directly accounts for the greater opportunity for significant variation over longer intervals and thus maximizes safety while keeping the glycemic outcome distribution best aligned in the desired range to maximise the opportunity for outcome BG in that range.

These initial results indicate that STAR is effective at reducing clinical effort, which has been a major drawback for TGC [20]. In particular, STAR reduced the number of measurements per day for all patients and the number of changes in intervention for most. Thus, over a larger study, STAR should reflect the savings in clinical burden from ~20% reductions in measurements (vs. SPRINT) and further savings from reduced numbers of changed interventions (more unchanged interventions).

From a broader human factors aspect, staff perception of workload is influenced by the number of measurements per day, actual time spent at the bedside performing measurements and administering treatment, and the quality of control obtained [24]. Thus, if a protocol is able to effectively regulate glycemic levels and achieve clinical outcomes, impressions of clinical staff are more positive and perceived effort is (at least slightly) reduced. Although STAR reduced measurements per day and other effort it is computer-based, which requires data entry and calculation run-time. As a paper-based protocol SPRINT, is faster in this respect and may be more transparent in its operation to users [24], which also affects perceived effort and compliance. Hence, perceptions of effort will likely hinge on the longer-term outcomes of clinical implementation.

Interestingly, in this initial study, nurses chose the 2-hour interval far more frequently than the (equally) available 3-hour interval. This outcome may reflect habit from using SPRINT, which has a maximum 2-hour interval, lack of familiarity or trust of the new system, or that the effort required was acceptable to nurses with the shorter interval.

One limitation of any model-based approach is the model and its ability to predict outcomes to interventions [28]. However, this model and related *in silico *methods have been extensively tested clinically [33,35-37,42] and validated for specific patients and in predicting both the median and variability of clinical trial outcomes, as well as for predicting specific intervention outcomes [23,43,44]. It is the only such model validated to this extent to date [34].

The STAR glycemic control approach presented is fully generalizable. The clinical targets and ranges can be set directly by clinical staff, as can the desired risk of hypo- or hyperglycemia (maximum 5% for BG < 4.0-4.4 mmol/L in Table 2). Hence, the approach is entirely flexible. The ranges and risk values used represent those chosen at Christchurch Hospital.

In contrast, whereas the glycemic ranges used in this study broadly match those in the design of SPRINT, SPRINT was fixed in its implementation and did not allow this flexibility and could not be adjusted directly by clinical staff for different patients or groups. This flexibility has been demonstrated for the STAR framework in ongoing pilot trials in Belgium [45]. As noted, two uses of STAR in the overall framework might yield very different glycemic outcomes due to: 1) different glycemic targets; 2) different choices of risk levels for the 5% lower glycemia bound; 3) different control intervention choices (insulin, nutrition, or both); 4) any specific clinical rules within the STAR approach that would modify the use of certain interventions, such as bolus or infusion insulin delivery; and 5) choice of glycemic limit of for 2- or 3-hourly measurements. As a result, this work is quite different from the use of STAR in [45], which uses fixed nutrition rates (nutrition is not used in control), delivers insulin via infusion rather than bolus delivery, has a higher (5.5 mmol/L) 5% lower glycemic threshold (vs. 4.0-4.4 mmol/L here), and thus a higher (5.5-8.0 mmol/L) desired glycemic band (vs. 4.0-6.5 mmol/L here). Thus, the comparison of these two works, as well as to SPRINT, clearly shows the flexibility of the overall STAR framework to deliver very different glycemic control approaches within the same stochastic, model-based approach, as well as the resulting ability to customize the TGC approach to meet local clinical standards, goals, and clinical workflow.

A further potential limitation of this overall STAR framework and approach is the stochastic model. Its forecasting is at the center of all the major advantages enabled by this approach. It also is a cohort-based model, which means that for some patients it will be too conservative, whereas for others potentially not conservative enough [32,45]. Equally, there is no guarantee that all ICU cohorts would have similar metabolic variability. However, these models can be readily created from existing clinical data for any reasonably similar metabolic system model [29,30,32]. Perhaps more importantly, a recent study found similar metabolic variability between NZ and Belgian ICU cohorts [23], although this specific result needs to be further generalized going forward.

Compliance and delays can be limitations of TGC studies. In this study, although not directly quantified, compliance to recommendations was very good. Equally, where STAR recommendations are overridden by nurses the system is told, as part of regular use, and thus it adapts by using that data for the next recommendation. Equally, delays are accounted for by the computerized system and thus do not really exist as a factor. Hence, the computerized approach enables delays to be tabulated without input and noncompliance to recommendations to be noted and accounted for in subsequent calculations, advantages that paper-based protocols do not offer.

Finally, this study is limited to the initial results showing performance and safety. Whereas patient numbers are limited, the overall hours of control is significant with more than 600 hours for critically ill patients. However, further studies [45] will provide evidence to the overall quality of the STAR framework in different uses, as well as its robustness to larger cohorts. These trials are ongoing internationally. However, although these results may not yet provide fully generalizable conclusions to guide therapy overall, they do serve to show initial safety and efficacy to justify extended use and trials.

Clinically, the comparison to the SPRINT results in Tables 5 and 6 yields insights relevant to the broader field. Specifically, whereas SPRINT was successful in providing safer and tighter control than most studies, it required 2-hourly measurements. These initial results clearly show that control can be achieved in measurement interval to 3-hourly, thus reducing clinical effort and burden, without reducing safety or efficacy. Second, the nutrition rates are much higher for these patients than for SPRINT, indicating that a model-based approach can achieve better control whilst providing more nutrition at the same time. Hence, the overall results can influence clinical thinking with respect to the measurement rates and nutrition levels from which good control might be still be achieved, where, in contrast, protocols with uncontrolled or unknown nutrition levels and 4-hourly or greater maximum measurement intervals [23,46,47] have not provided the same efficacy or safety as this initial study and SPRINT.

## Conclusions

This research presents the initial pilot trial results for a novel Stochastic TARgeted (STAR) TGC framework and approach. The results show that this approach can provide quality control performance that is tighter across patients and thus more patient-specific. Equally, it also reduced light hypoglycemia using a clinically specified maximum risk with stochastic forecasting of metabolic variation, as well as significantly reducing clinical workload compared with the current clinical standard protocol at Christchurch Hospital. The stochastic forecasting is unique in this field and enables a maximum likelihood approach to targeting a desired glycemic range while enabling the clinical risk of hypo- or hyperglycemia to be directly managed. It also enables patients with very different metabolic (intra- and inter- patient) variability to be directly managed and controlled within a single (STAR) model-based framework.

More specifically, the STAR approach presented is fully generalizable and clinical targets and ranges can be set directly by clinical staff, with those used here representing those chosen at Christchurch Hospital. These initial results remain to be proven over subsequent clinical pilot trials ongoing toward a potential transition to regular clinical practice implementation.

## Competing interests

The authors declare that they have no competing interests.

## Authors' contributions

All authors were involved in developing the STAR concept and methods. Clinical trials were implemented by GMS in the Christchurch ICU. Software and systems for the trials were created by AE, JS, CST, LW and ALC with input from all other authors. Data was gathered and analysed by AE, JS, CST, LW, JGC and ALC. The manuscript was originally drafted by AE, JS, CST, LW, JGC and ALC, but all authors made contributions through the entire process, including reading and final approval.

## Supplementary Material

Additional file 1 **Appendix: Metabolic System Model**. Click here for file
