# Supplementary material for: Pilot proof of concept clinical trials of Stochastic Targeted (STAR) glycemic control
Source: Ann Intensive Care. 2011 Sep 19;1:38. doi: 10.1186/2110-5820-1-38 (PMC3224394; doi:10.1186/2110-5820-1-38)
Supplement: Additional file 1 — Appendix: Metabolic System Model. [file 2110-5820-1-38-S1.DOC]

**ADDITIONAL FILE 1:**

**APPENDIX: Metabolic System Model**

The metabolic system model used has been extensively validated in virtual and clinical trials and is defined [33, 34, 38]:


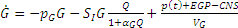
 (1)


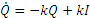
 (2)


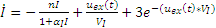
 (3)

Where:


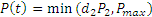
 (4)


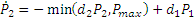
 (5)


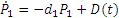
 (6)

All model parameters are described in Table A-1, including values for the population constants employed. Importantly, the value of insulin sensitivity, SI(t), is identified hourly based on clinical measurements at the bedside. Details on identifying SI(t) are found in previous studies [29, 44]. The mathematics and details on how to create the stochastic models of insulin sensitivity are detailed in references 29 and 30.

**Table A-1. Variable definitions and values for the glucose-insulin system model of Equations (1)-(6)**

| **Variable** | **Description** | **Values** |
| --- | --- | --- |
| *G* | Blood glucose level | (mmol/L) |
| *p_G_* | Insulin independent glucose removal (excluding central nervous system uptake) and the suppression of *EGP* from *EGP_b_* with respect to *G* | 0.006 (min^−1^) |
| *α_G_* | Saturation parameter for insulin mediated glucose removal | 1/65 (L/mU) |
| *S_I_* | Insulin mediated glucose removal and the suppression of *EGP* from *EGP_b_* with respect to *G* and *Q* | (L/mU/min) |
| *Q* | Interstitial insulin concentration | (mU/L) |
| *P*(*t*) | Glucose appearance in plasma from dextrose intake | (mmol/min) |
| *EGP* | Endogenous glucose production | 1.16 (mmol/min) |
| *CNS* | Central nervous system glucose uptake | 0.3 (mmol/min) |
| *V_G_* | Plasma glucose distribution volume | 13.3 (L) |
| *K* | Interstitial insulin transport rate | -ln (0.5)/35 (min^−1^) |
| *I* | Plasma insulin concentration | (mU/L) |
| *N* | Plasma insulin decay rate | 0.16 (min^-1^) (min^−1^) |
| *α_I_* | Saturation parameter for plasma insulin clearance | 1.7 x 10^-3^ (L/mU) |
| *u_ex_*(*t*) | Exogenous insulin | (mU/min) |
| *V_I_* | Plasma insulin distribution volume | 3.15 (L) |
| *d*_2_ | Glucose absorption rate from gut | -ln (0.5)/100 (min^−1^) |
| *P*2 | Glucose level in gut | (mmol) |
| *P*_max_ | Maximal glucose flux from gut to plasma | 6.11 (mmol/min) |
| *P1* | Glucose level in stomach | (mmol) |
| *d*_1_ | Glucose absorption rate from stomach | -ln (0.5)/20 (min^−1^) |
| *D*(*t*) | Dextrose intake | (mmol/min) |
